# Maternal Prenatal Depressive Symptoms and Fetal Growth During the Critical Rapid Growth Stage

**DOI:** 10.1001/jamanetworkopen.2023.46018

**Published:** 2023-12-04

**Authors:** Lu Zhang, Ping Li, Qiaoyue Ge, Zeyuan Sun, Jiarui Cai, Chenghan Xiao, Chuan Yu, Chiara Nosarti, Jiaqiang Liao, Zhenmi Liu

**Affiliations:** 1 Department of Maternal and Child Health, Child and Adolescent Health, West China School of Public Health and West China Fourth Hospital, Sichuan University, Chengdu, China; 2 Institute of Systems Epidemiology, West China School of Public Health and West China Fourth Hospital, Sichuan University, Chengdu, China; 3 Department of Epidemiology and Biostatistics, West China School of Public Health and West China Fourth Hospital, Sichuan University, Chengdu, Sichuan, China; 4 Department of Child and Adolescent Psychiatry, Institute of Psychiatry, Psychology and Neuroscience, King’s College London, United Kingdom; 5 Centre for the Developing Brain, School of Biomedical Engineering & Imaging Sciences, King’s College London, United Kingdom

## Abstract

**Importance:**

Fetal growth in the critical rapid growth stage (CRGS) before delivery, approximately between 30 to 37 gestational weeks, carries significant implications for subsequent overweight, obesity, and arterial health. Previous evidence has demonstrated the association between maternal depressive symptoms and fetal growth trajectories from early to late pregnancy, but there remains limited understanding of the association of these symptoms with the longitudinal fetal growth change within the CRGS.

**Objective:**

To investigate the association between maternal depressive symptoms and fetal growth during the CRGS before delivery.

**Design, Setting, and Participants:**

This prospective birth cohort study was conducted from January 2018 to December 2020. Volunteer pregnant women were enrolled in their first trimester of prenatal visits. Women with severe disease before pregnancy and multiple births, fetuses with congenital anomalies, and preterm or postterm births were excluded. This multicenter study was based in 13 hospitals covering 81 counties across 12 cities in Sichuan Province, China. Follow-up visits were performed at the second trimester, the third trimester, and 24 hours after delivery. The analysis was conducted from January to May 2023.

**Exposures:**

Maternal depressive symptoms, as a continuous variable, measured by the Edinburgh Postpartum Depression Scale (EPDS) at a median gestational week of 24 (range, 14 to 27) weeks of gestation. A higher score on the EPDS indicates worse depressive symptoms.

**Main Outcomes and Measures:**

The main outcomes included ultrasonography-measured biparietal diameter (BPD), femur length (FL), and abdominal circumference (AC), along with calculated estimated fetal weight (EFW). These parameters were evaluated longitudinally at a median gestational week of 30 (range, 28 to 32) and 37 (range, 35 to 39) weeks. Linear mixed models were used to estimate the associations between maternal depressive symptoms and fetal growth parameters.

**Results:**

A total of 2676 mother-offspring dyads were included, in which the mean (SD) age of mothers was 28.0 (4.4) years, and 1294 (48.4%) of the offspring were female. The median (IQR) maternal EPDS score was 5.0 (4.0 to 9.0). After adjustment for confounders, a significant correlation was found between a higher score of depressive symptoms in mothers and a slower rate of fetal growth across FL (β = −0.40; 95% CI, −0.58 to −0.22), AC (β = −1.97; 95% CI, −2.90 to −1.03), and EFW (β = −50.11; 95% CI, −68.46 to −31.75). These associations were stronger in female fetuses or those with better family socioeconomic conditions.

**Conclusions and Relevance:**

In this prospective cohort study, maternal depressive symptoms were associated with slower fetal growth rate in the CRGS before delivery. Early screening for depressive disorders in pregnant women appears to be essential for fetal growth and later health.

## Key Points

**Question:**

Are maternal depressive symptoms associated with the rate of fetal growth in the critical rapid growth stage before delivery?

**Findings:**

In this prospective cohort study including 2676 mother-offspring dyads, higher scores of depressive symptoms in mothers were significantly associated with a slower fetal growth rate for femur length, abdominal circumference, and estimated fetal weight between 30 to 37 gestational weeks.

**Meaning:**

Maternal depressive symptoms during pregnancy may be associated with slower fetal growth rate in the critical rapid growth stage before delivery, emphasizing the importance of screening and intervention for mental disorders during pregnancy.

## Teaser

This cohort study investigates the association between maternal depressive symptoms and fetal growth during the critical rapid growth stage before delivery.

## Introduction

Intrauterine development represents a sensitive window strongly associated with subsequent lifetime health risks.^1,2^ Between approximately 30 and 37 gestational weeks,^3,4,5^ there is a critical period of rapid fetal growth that we refer to as the critical rapid growth stage (CRGS) before delivery. Evidence has documented that the velocity of estimated fetal weight and formulation of adipose tissue change the most during this stage,^6,7,8^ contributing to newborns’ adaptation to the thermal and nutritional challenges after birth. This critical stage also plays a crucial role in the development and maturation of fetal white matter,^9,10,11^ with synapse formation peaking at 34 weeks of gestation,^12^ playing a fundamental role in shaping early neural circuits.^13^ Due to the essential role of CRGS, disrupted fetal growth during this stage has complex and profound implications for offspring health, which have been shown to be associated with early markers of impaired arterial health^14^ as well as increased overweight and obesity risk at age 4 years.^15^ However, understanding of the risk factors associated with fetal growth during this critical stage remains limited.

Prenatal depression is a common complication experienced by pregnant women, exhibiting a pooled prevalence of approximately 20%, particularly pronounced in low-income and middle-income countries.^16,17,18^ Evidence from the Generation R study has revealed negative associations of maternal depressive symptoms with growth rates of fetal head circumference and fetal weight throughout early to late pregnancy.^19^ Yet, another cohort study also examined the association using fetal growth curves but found no significant result.^20^ These studies focused on fetal growth trajectories throughout the entire pregnancy, and only 1 view of fetal growth at the third trimester of pregnancy was collected, possibly overlooking critical information on fetal growth changes during the CRGS. As far as we know, there is a paucity of evidence utilizing longitudinal measurements during the CRGS before delivery to explore the potential association between maternal depressive symptoms and fetal growth. Moreover, it is important to acknowledge the substantial racial and ethnic variations in fetal growth.^21^ Unfortunately, most existing studies were conducted in Western countries,^19,20^ thus restricting the generalizability of these findings to East Asia. Therefore, based on a multi-ethnic Chinese birth cohort, we aimed to examine the associations of maternal depressive symptoms with longitudinally measured fetal growth parameters during late pregnancy.

## Methods

### Study Population

This birth cohort study was established between January 2018 and December 2020, encompassing 81 counties across 12 cities in Sichuan Province, China (eFigure 1 in Supplement 1). Pregnant women who attended their first prenatal visits between 6 and 13 weeks of gestation at the 1 of 13 study hospitals were eligible. Women were enrolled in the birth cohort study if they (1) intended to complete the pregnancy examinations and delivery at studied hospital; (2) agreed to have follow-up interviews; and (3) had no severe mental illness, cognitive dysfunction, or other conditions impeding the completion of the investigation. Follow-up visits were conducted at the second trimester, the third trimester, and 24 hours after delivery. The demographic, socioeconomic, and behavioral characteristics were collected through face-to-face questionnaires. Disease histories and complications of pregnancy were collected through both self-report and medical records. All participants provided written informed consent prior to enrollment. The study proposal was approved by the Ethics Committee of West China Forth Hospital and West China School of Public Health, Sichuan University. This report follows the Strengthening the Reporting of Observational Studies in Epidemiology (STROBE) reporting guideline for cohort studies.

In this study, we initially included 10 524 pregnant women who had complete information at baseline. To establish the prospective temporal association between exposures and outcome, we restricted the time of maternal symptom measurements to the second trimester of pregnancy (14-27 weeks of gestation), and ultrasonography measurements at 30 ± 2 and 37 ± 2 weeks of gestation, which excluded 3803 and 3905 participants separately. Then, to maximally generalize the study associations, we further excluded 140 participants with multiple births, infants with congenital anomalies, postterm deliveries, and deliveries with missing times. Finally, 2676 participants were included in our study (eFigure 2 in Supplement 1).

### Exposure and Measurement

Exposure was defined as having maternal depressive symptoms during the second trimester (median [IQR], 24.0 [23.0-25.0] weeks) assessed by the Edinburgh Postpartum Depression Scale (EPDS).^22^ The EPDS is a validated screening scale for depressive symptoms in postpartum women, but it is also widely applied during pregnancy.^23,24^ The validity of its implementation in mainland China has already been well-documented.^25^ This scale consists of 10 items, each scored on a 4-point Likert-scale from 0 to 3, and the total score ranges from 0 to 30 points, with 0 being the minimum and 30 the maximum score. A higher EPDS score indicates more severe depressive symptoms.

### Outcomes

Fetal growth outcomes included biparietal diameter (BPD), femur length (FL), abdominal circumference (AC), and estimated fetal weight (EFW). BPD, FL, and AC were measured in centimeters by professional and licensed sonographers at 30 ± 2 weeks and 37 ± 2 weeks of gestational age and converted into millimeters for analysis. EFW was estimated according to the Hadlock algorithm and measured in grams.^26^ To ensure reliability and consistency across institutions, we provided standardized training for our sonographers before performing operations. Furthermore, we constructed gestational age–adjusted SD scores for each fetal growth parameter to correct the gestational age influence, which were performed as a sensitivity analysis (eMethods 1 and eTable 3 in Supplement 1). Gestational age at each ultrasonography measurement (GAUM) was calculated as the date of measurement minus the date of last menstrual period if it agreed with the ultrasonography-corrected gestational age within 7 days, otherwise the ultrasonography-corrected gestational age was used.

### Covariates

A range of potential confounders, including demographic characteristics and lifestyle factors, were considered. Maternal age was categorized as less than 25 years, 25 to 30 years, or greater than 30 years. Ethnic minority area was dichotomized as yes or no according to a list of ethnic minority autonomous regions published by the government of Sichuan Province, China.^27^ Maternal education level was categorized as junior school and less, senior school, junior college, or university and higher based on the Chinese education system. Household annual income was categorized as less than CNY 60 000, CNY 60 000 to CNY 99 999, or CNY 100 000 or more (CNY 7.25 is approximately $1). Maternal prepregnancy body mass index (BMI) was calculated as weight in kilograms divided by height in meters squared. Parity was dichotomized as primipara and multipara. Folic acid supplement before pregnancy was dichotomized as yes or no. All this information was collected at baseline by the mother self-reporting. Maternal passive smoking was defined as passive inhalation of cigarette smoke by smokers for at least 15 minutes a day and more than 1 day a week. Alcohol consumption was defined as drinking at least once a week. Information on passive smoking and alcohol consumption was collected at baseline and in the third trimester of pregnancy. If the answer to both surveys was negative, then the final answer was taken as no; otherwise, it was taken as yes. Healthy eating score was calculated using diet data from a semiquantitative validated food frequency questionnaire with reference to the alternative Health Eating Index–2010^28^ and was divided into 5 quintiles, with higher quintiles representing a better diet (eMethods 2 in Supplement 1).

### Statistical Analysis

The distributions of maternal depressive symptom scores stratified by demographic and socioeconomic characteristics and behavioral factors were summarized by mean and SD. The significance of these differences was tested by *t* test or 1-way analysis of variance. For each fetal growth parameter, we estimated the mean differences in fetal growth rate for each IQR increase in maternal EPDS scores using a product interaction term between maternal EPDS score and GAUM, based on linear mixed regression models with a random intercept at the individual level following the Gaussian distribution to account for the nonindependence of repeated measurements. Additionally, we allowed more complex assumptions in the linear mixed models, which included a random slope of GAUM at individual levels to account for different growth rates between individuals. Differences of these models were compared using likelihood ratio tests. Furthermore, to examine the potential nonlinear associations underlying the study associations, we replaced the continuous maternal EPDS scores by quintiles for analysis (reference level, the lowest quintile). The significance of *P* for trend was tested by including the median value of maternal depressive score within each quintile stratum as a continuous variable in regression models. In multivariable-adjusted regression analyses, we used the directed acyclic graphs methods to select the potential confounders^29^ (eFigure 3 in Supplement 1). Additionally, we adjusted for confounders on the fetal growth trajectories by adding interaction terms between the confounders, which maintained statistical significance on fixed-effects analyses, and GAUM. Imputation of missing covariate data was conducted using the multivariate imputation by chained equations.^30^

We conducted several stratified analyses to evaluate modifications. We first replicated the main analyses stratified by maternal educational levels and annual household income to evaluate the socioeconomic variations on the study associations. As different ethnic subgroups in China represent different genetic and lifestyle characteristics,^31^ we also replicated the main analyses stratified by ethnic residential areas. Given that evidence indicates the maternal inflammation status is associated with fetal growth,^32^ we also conducted stratified analyses by prepregnancy BMI and healthy eating score categories. We finally tested the difference in the study associations by fetal sex to better understand sex differences in fetal growth.^33^ *P* for interaction was calculated to examine the significance of the modifications by using the likelihood ratio tests to compare the full model, which included the interaction term, with the reduced model, which did not include that interaction term.

Several sensitivity analyses were conducted to assess the reliability of our findings. First, considering the difference in GAUM, we repeated our main analyses using gestational age–adjusted SD score as dependent variables. Then, acknowledging that some metabolic disorders before and during pregnancy may bias our estimates, we performed analyses by excluding participants with hyperthyroidism, hypothyroidism or other serious diseases before pregnancy, or those with gestational diabetes (GD) or hypertensive disorders of pregnancy (HDP). Finally, we also excluded participants with missing covariates to replicate the analyses.

Results were presented as beta estimates and their 95% CIs. Benjamini-Hochberg false-discovery rate (FDR) correction was used to account for the multiple comparisons.^34^ All analyses were performed using R version 4.1.1 (R Project for Statistical Computing), using 2-tailed test with *P* < .05 indicating statistical significance.

## Results

### Characteristics

A total of 2676 mother-infant pairs were included in the overall analytic sample. Mothers had a mean (SD) age of 28.0 (4.4) years, and 918 (34.3%) of them were older than 30 years. More than half (54.5%) of mothers were primiparous. Among their infants, there are more males than females (1382 [51.6%] vs 1294 [48.4%]). Maternal depressive symptoms were measured during 14 to 27 weeks of gestation, with a median (IQR) examination time of 24 (23-25) weeks of gestation. The median (IQR) maternal EPDS score was 5.0 (4.0-9.0). Maternal EPDS scores exhibited significant differences in relation to maternal age, educational level, annual household income, ethnic minority area, healthy eating score, passive smoking, and GD groups (Table 1). The median times of the 2 ultrasonography measurements were 30 and 37 weeks, respectively. The mean and SD of each fetal growth parameter are represented in Table 2.

**Table 1.** Baseline Characteristics of the Study Participants

Characteristic	Participants, No. (%) (N = 2676)	EPDS score, mean (SD)	*P* value^a^
Age at recruitment, y			
<25	610 (22.80)	7.06 (3.77)	.02
25-30	1148 (42.90)	6.80 (3.73)	
≥30	918 (34.30)	6.53 (3.76)	
Mean (SD)	27.98 (4.38)	NA	NA
Maternal education level			
Junior school and less	607 (22.68)	7.29 (4.02)	<.001
Senior school	859 (32.10)	6.96 (3.63)	
Junior college	750 (28.03)	6.52 (3.78)	
University and higher	460 (17.19)	6.11 (3.46)	
Annual household income, CNY			
<60 000	1136 (42.45)	7.27 (3.88)	<.001
60 000-99 999	1039 (38.83)	6.40 (3.55)	
≥100 000	501 (18.72)	6.38 (3.74)	
Ethnic minority area^b^			
No	2311 (86.36)	6.35 (3.64)	<.001
Yes	365 (13.64)	9.42 (3.39)	
Prepregnancy BMI			
<18.5	484 (18.09)	7.12 (3.91)	.07
18.5-23.9	1744 (65.17)	6.68 (3.70)	
≥24	448 (16.74)	6.72 (3.77)	
Parity			
Primipara	1458 (54.48)	6.69 (3.69)	.26
Multipara	1218 (45.52)	6.86 (3.83)	
Folic acid supplement			
No	1784 (66.67)	6.81 (3.86)	.44
Yes	892 (33.33)	6.69 (3.53)	
Healthy eating score quintile^c^			
1, Lowest	568 (21.23)	6.53 (3.55)	<.001
2	433 (16.18)	6.96 (3.78)	
3	499 (18.65)	7.83 (4.01)	
4	551 (20.59)	6.19 (3.57)	
5, Highest	625 (23.36)	6.52 (3.71)	
Mean (SD)	36.43 (5.52)	NA	NA
Maternal passive smoking			
No	2493 (93.16)	6.70 (3.73)	.002
Yes	183 (6.84)	7.67 (3.95)	
Maternal alcohol consumption			
No	2639 (98.62)	6.76 (3.74)	.28
Yes	37 (1.38)	7.54 (4.49)	
Gestational diabetes			
No	2476 (92.53)	6.83 (3.76)	.001
Yes	200 (7.47)	5.98 (3.53)	
Hypertensive disorders complicating pregnancy			
No	2652 (99.10)	6.78 (3.75)	.06
Yes	24 (0.90)	5.21(3.80)	
Neonatal sex			
Male	1382 (51.64)	6.78 (3.74)	.83
Female	1294 (48.36)	6.75 (3.77)	

Abbreviations: BMI, body mass index (calculated as weight in kilograms divided by height in meters squared); EPDS, Edinburgh Postnatal Depression Scale; NA, not applicable.

^a^ Tests were conducted using *t* test or 1-way analysis of variance, with maternal EPDS score as the dependent variable.

^b^ According to a list of ethnic regions published by the government of Sichuan Province, China.

^c^ The intake frequency of each food category was divided into quintiles, with healthy foods scored from 0 to 4, and unhealthy foods scored from 4 to 0. The healthy eating score is the sum of all food categories’ scores and classified by quintiles.

**Table 2.** Distributions of Fetal Growth Parameters at Different Gestational Ages

Fetal growth parameter	Mean (SD)	
	30 ± 2 wk	37 ± 2 wk
Biparietal diameter, mm	78.25 (4.14)	92.28 (3.22)
Femur length, mm	58.34 (3.30)	71.44 (2.64)
Abdominal circumference, mm	265.25 (14.62)	333.77 (15.19)
Estimated fetal weight, g	1655.72 (247.47)	3180.95 (342.10)

### Maternal Depressive Symptoms and Fetal Growth Rate

**Figure 1.**
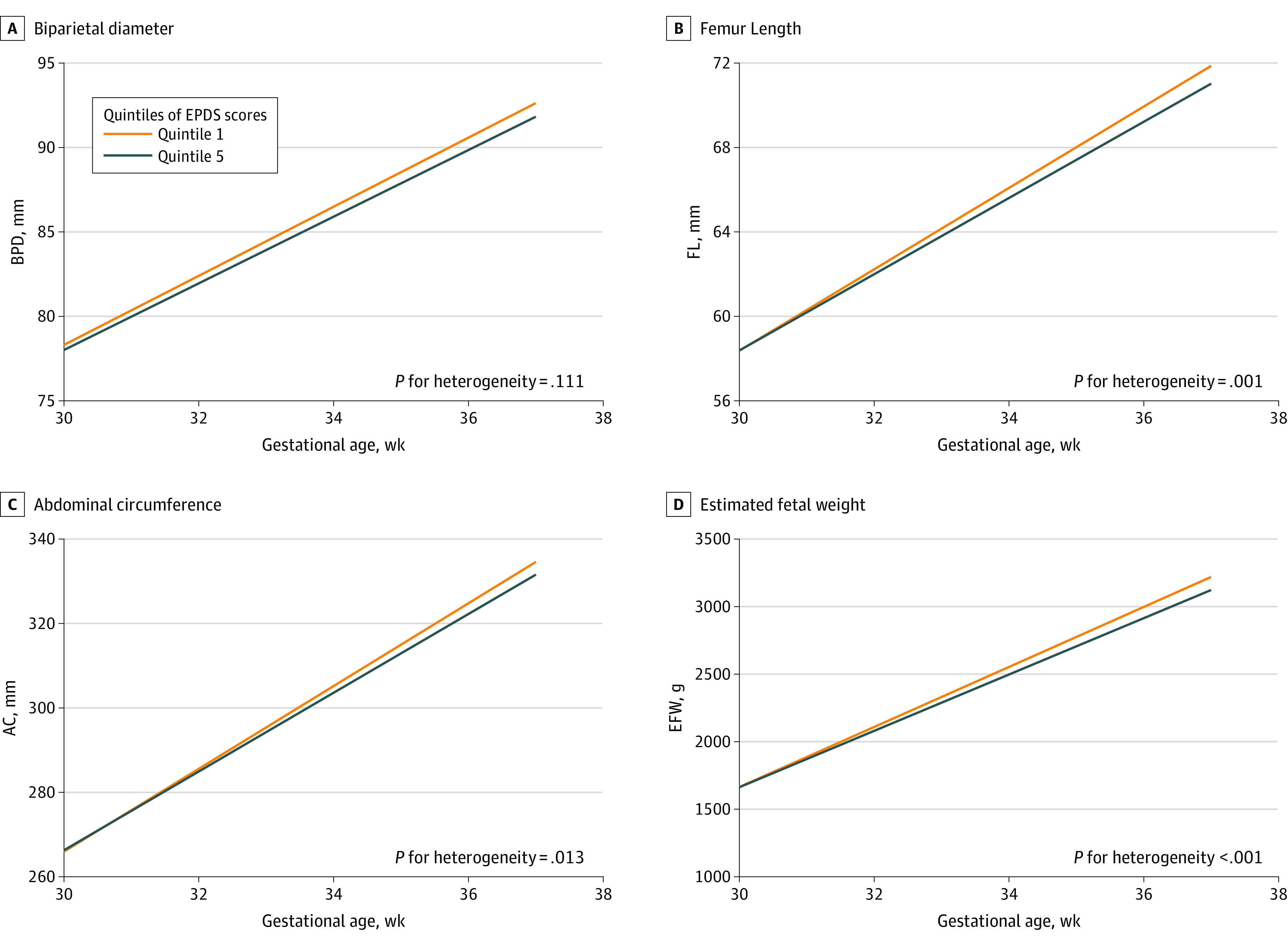
Edinburgh Postnatal Depression Scale (EPDS) and Fetal Growth *P* for heterogeneity indicated the significance of gestational age at each ultrasonography measurement × quintiles of maternal depressive status interaction term. AC indicates abdominal circumference; BPD, biparietal diameter; EFW, estimated fetal weight; FL, femur length.

A significant difference was observed between models incorporating and excluding a GAUM random slope for BPD, FL, and EFW (eTable 1 in Supplement 1). For consistency, we included all growth parameters in this study with a random slope of GAUM. In crude models, each IQR increase in maternal EPDS score was significantly associated with a slower growth rate of FL (β = −0.40; 95% CI, −0.58 to −0.22; FDR-corrected *P* < .001), AC (β = −1.97; 95% CI, −2.90 to −1.03; FDR-corrected *P* < .001), and EFW (β = −50.11; 95% CI, −68.46 to −31.75; FDR-corrected *P* < .001) but not with BPD (β = −0.21; 95% CI, −0.42 to 0.01; FDR-corrected *P* = .11). After adjusting for potential confounders, these associations remained largely unchanged (Table 3). Furthermore, growth trajectories across quintiles of maternal EPDS scores indicated that fetuses of mothers in the highest quintile of depressive status experienced significantly decreased growth rate from gestational weeks 30 to 37 compared with those in the lowest quintile, except for BPD (eg, EFW: β = −90.56; 95% CI, −138.53 to −42.59) (Figure 1; eTable 2 in Supplement 1).

**Table 3.** Association of 1-IQR Increase in EPDS Score With Fetal Growth

Fetal growth parameter	EPDS score, crude β (95% CI)	*P* value^a^	GAUM × EPDS score, crude β (95% CI)	*P* value^a^	EPDS score, adjusted β (95% CI)^b^	*P* value^a^	GAUM × EPDS score, adjusted β (95% CI)^b^	*P* value^a^
Biparietal diameter, mm	−0.18 (−0.39 to 0.03)	.17	−0.23 (−0.44 to −0.02)	.07	−1.04 (−2.40 to 0.31)	.21	−0.21 (−0.42 to 0.01)	.11
Femur length, mm	−0.01 (−0.18 to 0.16)	.90	−0.42 (−0.60 to −0.24)	<.001	−0.82 (−1.99 to 0.35)	.22	−0.40 (−0.58 to −0.22)	<.001
Abdominal circumference, mm	0.42 (−0.31 to 1.16)	.30	−2.08 (−3.01 to −1.14)	<.001	−3.55 (−9.57 to 2.48)	.30	−1.97 (−2.90 to −1.03)	<.001
Estimated fetal weight, g	1.47 (−11.03 to 13.97)	.87	−52.32 (−70.67 to −33.97)	<.001	−85.40 (−203.54 to 32.74)	.22	−50.11 (−68.46 to −31.75)	<.001

Abbreviations: EPDS, Edinburgh Postnatal Depression Scale; GAUM, gestational age at ultrasonography measurement.

^a^ *P* values after false-discovery rate corrections.

^b^ Adjusted for maternal age, ethnic area, maternal education level, household annual income, maternal prepregnancy weight status, parity, folic acid supplement before pregnancy, maternal passive smoking, maternal alcohol consumption, healthy eating score, GAUM, and interaction terms between GAUM and passive smoking and alcohol consumption.

### Subgroup Analyses

**Figure 2.**
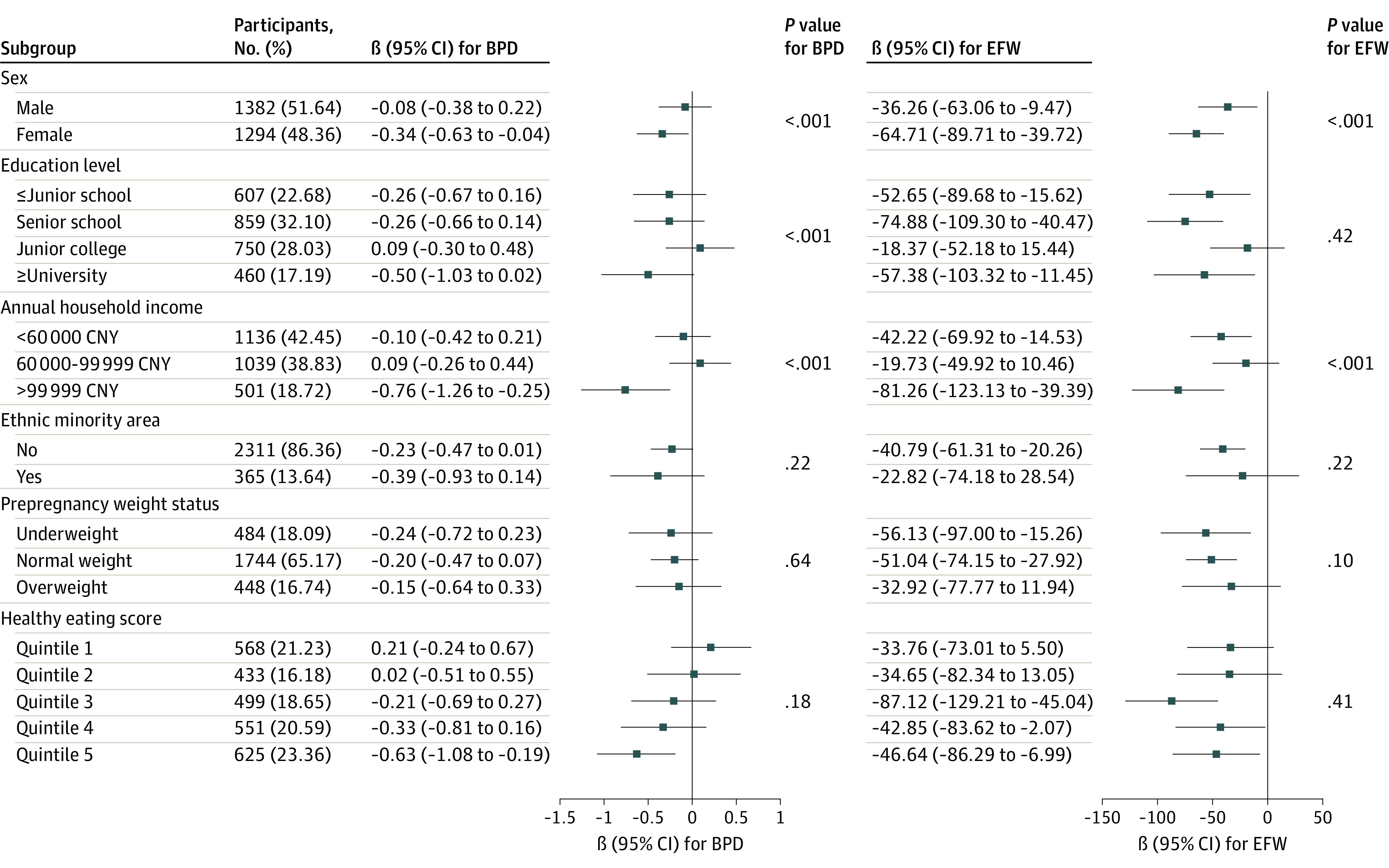
Subgroup Analyses of Edinburgh Postnatal Depression Scale (EPDS) and Fetal Growth *P* value indicates the significance of the gestational age at each ultrasonography measurement × EPDS scores × subgroup categories interaction term. BPD indicates biparietal diameter; EFW, estimated fetal weight.

In terms of stratification by fetal sex, the association was significantly different between male and female subgroups (eg, BPD: β = −0.08; 95% CI, −0.38 to 0.22; vs β = −0.34; 95% CI, −0.63 to −0.04; *P* for interaction < .001), especially more pronounced for female fetuses. Moreover, annual household income significantly modified the association between maternal EPDS score and growth rates of BPD, FL, AC, and EFW (eg, BPD: <CNY 60 000, β = −0.10; 95% CI, −0.42 to 0.21; CNY 60 000 to 99 999, β = 0.09; 95% CI, −0.26 to 0.44; >CNY 99 999, β = −0.76; 95% CI, −1.26 to −0.25; *P* for interaction < .001). Notably, the associations were potentiated in fetuses from higher income families or those with higher maternal educational levels. For the stratification by ethnic minority areas, significant modification effect was only found on EFW. However, we detected no evidence suggesting that prepregnancy weight status or healthy eating score modified the association between maternal EPDS scores and fetal growth rates (Figure 2; eFigure 4 in Supplement 1).

### Sensitivity Analyses

Similar to the main results, sensitivity analysis using gestational age–adjusted SD scores revealed that maternal EPDS score was negatively associated with fetal growth rate of FL and EFW (eTable 4 in Supplement 1). After excluding participants with maternal metabolic disorders before or during pregnancy, our results remained largely unchanged (eTables 5-7 in Supplement 1). These observations for FL and EFW remained consistent even after excluding participants with incomplete covariates (eTable 8 in Supplement 1).

## Discussion

In this prospective cohort study, we provided evidence associating maternal prenatal depressive symptoms with decelerated fetal growth rates of FL, AC, and EFW between 30 to 37 gestational weeks. The associations remained significant after adjusting for maternal demographic characteristics, socioeconomic characteristics, behavioral factors, and diet. Importantly, we found that the reduction in fetal growth rate was more pronounced in female fetuses or those born to wealthier families. To our knowledge, this study is the first East Asian cohort study to examine the associations between maternal depressive symptoms and fetal growth rate, with a particular emphasis on the CRGS during the late pregnancy.

The discovery of the association between higher maternal depressive symptom scores and slowed fetal growth aligns with extant evidence. Several studies have reported the associations between maternal depressive symptoms and increased risk of offspring low birth weight,^35^ smaller midbrain volumes,^36^ and decreased creatine and choline levels in fetal brain.^37^ Similarly, evidence from the Generation R study have found that maternal depressive symptoms were negatively associated with fetal head growth and fetal weight gain from the early to late pregnancy.^19^ In present study, we have expanded on these findings to FL and AC in the CRGS before delivery. Given the importance of this developmental stage for childhood cognitive function^10^ and cardiovascular health^14,15^ in later life, our study provides valuable insights into a potential pathway by which maternal depressive symptoms can contribute to adverse health outcomes via late-pregnancy fetal growth restriction.

There are several potential mechanisms that might account for the influence of maternal depressive symptoms on fetal growth. First, both animal and human researches have shown that the hypothalamic-pituitary-adrenal (HPA) axis plays an essential role in the association between maternal depressive symptoms and fetal growth, especially brain development.^38,39,40,41^ Stress exposure can trigger abnormal elevations in maternal cortisol levels, potentially leading to higher fetal cortisol levels and affecting fetal growth.^42^ Second, prenatal depressive symptoms may also increase the release of stress hormones, such as catecholamine, which could impair fetal growth by decreasing uterine blood supply and then restrict fetal growth.^43,44^ Additional potential mechanisms include oxidative stress, microbiome, immune system and inflammation.^45,46^

Our observation of stronger associations in female fetuses aligns with previous studies. A systematic review suggested that, compared with males, females have higher levels of HPA axis activity and placental glucocorticoid permeability when exposed to depression or stress.^47^ This could result in female fetuses being more sensitive to maternal depressive symptoms. Furthermore, similar sex-based differences have been reported in associations between exposure to prenatal maternal distress and elevated risks of childhood psychopathology^48^ and cardiovascular diseases.^49^

The stronger associations of maternal depressive symptoms with slower fetal growth rate were observed in fetuses from families with higher socioeconomic status. This pattern was similar with another study that work-life conflict had a stronger association with poor mental health for individuals with high socioeconomic status.^50^ One possible explanation for our finding is that more office work and sedentary behaviors and less physical activity in the population with high socioeconomic status,^51,52^ which might lead to an amplification of pathological changes in fetal growth associated with maternal depressive symptoms.^53,54,55^

Our findings support the health benefit for fetal growth and future health, by early screening of pregnant women with higher exposure to prenatal depressive symptoms, especially those with higher socioeconomic status or carrying a female fetus. More importantly, growth and developmental outcomes are not predetermined at birth, they are also influenced by postnatal care^56^ and behavioral establishment.^57,58^ Therefore, early intervention may be warranted for these children.

There are several strengths of our research. First, we focused on the repeated ultrasonography measurements of fetal growth parameters in CRGS with prospectively measured maternal depressive status, enhancing the ability to demonstrate causality. Second, many confounders were adjusted, which increased the credibility of the study associations. Finally, we brought important evidence for East Asia populations, reducing the external validity limitations^59^ of earlier studies conducted primarily in European and US contexts.^19,20^

### Limitations

Our study also has some limitations. First, when using fetal ultrasonography measurements, measurement errors are inevitable, although we minimized the errors by standardized training for sonographers. Second, we did not collect information on antidepressants or sleep medication usage, which could potentially bias the study associations. However, given that no study participants reported a medical diagnosis of prenatal psychiatric disorders, such as depression and schizophrenia, the likelihood of this occurring is not great. Third, our data inevitably suffer from missing data and selection bias to some degree. Nonetheless, the sensitivity analyses demonstrated the robustness of our findings. Additionally, as we did not include preterm or postterm births, the generalization of the study conclusions should be cautious in these populations.

## Conclusions

In conclusion, our study provides new evidence highlighting negative associations between maternal prenatal depressive symptoms and fetal growth rate in a CRGS before delivery. These findings underscore the importance of early detection and management of maternal depressive symptoms during pregnancy, particularly among women with higher socioeconomic status or female fetuses.

## Notes

Supplement 1. **eFigure 1.** Spatial Distribution of Baseline Populations Involved in the Cohort Study **eFigure 2.** Flowchart for the Study Participants Selection **eFigure 3.** The Constructed Directed Acyclic Graph for the Potential Confounders **eFigure 4.** Results of Subgroup Analyses for the Associations Between EPDS Scores (per IQR Increase) With Fetal FL and AC Growth Rates **eTable 1.** Likelihood Ratio Test for Adjusted Models With and Without Random Slope of Gestational Age at Ultrasonography Measurement **eTable 2.** Results of the Multivariable Adjusted Analyses of Quintiles of Maternal EPDS Score With Fetal Growth Rates **eTable 3.** Akaike Information of the Generalized Additive Models for Location, Scale, and Shape **eTable 4.** Associations of EPDS Score (per IQR Increase) With SD Scores of Fetal Growth Parameters **eTable 5.** Associations Between EPDS Score (per IQR Increase) With Fetal Growth Rate, After Excluding Participants With Gestational Diabetes or Hypertensive Disorders of Pregnancy **eTable 6.** Associations Between EPDS Score (per IQR Increase) With Fetal Growth Rate, After Excluding Participants With Severe Disease Before Pregnancy **eTable 7.** Associations Between EPDS Score (per IQR Increase) With Fetal Growth Rate, After Excluding Participants With Severe Disease Before Pregnancy, Gestational Diabetes, or Hypertensive Disorders of Pregnancy **eTable 8.** Associations Between EPDS Score (per IQR Increase) With Fetal Growth Rate, Without Imputation of Missing Covariates **eMethods 1.** Calculation for Gestational Age–Adjusted SD Scores **eMethods 2.** Calculation for Healthy Eating Scores in Our Study

Supplement 2.
Data Sharing Statement
