# Error in Affiliation

**DOI:** 10.1001/jamanetworkopen.2023.54165

**Published:** 2024-01-08

In the Original Investigation titled “Maternal Prenatal Depressive Symptoms and Fetal Growth During the Critical Rapid Growth Stage,”^1^ published December 4, 2023, there was an error in the author affiliations. The affiliation for Dr Zhang, Mr Li, Ms Ge, Dr Cai, Dr Xiao, Dr Yu, Dr Liao, and Dr Liu should have been Department of Maternal, Child and Adolescent Health, West China School of Public Health and West China Fourth Hospital. This article has been corrected.^1^
