# Supplementary material for: Maternal Prenatal Depressive Symptoms and Fetal Growth During the Critical Rapid Growth Stage
Source: JAMA Netw Open. 2023 Dec 4;6(12):e2346018. doi: 10.1001/jamanetworkopen.2023.46018 (PMC10696489; doi:10.1001/jamanetworkopen.2023.46018)
Supplement: Supplement 1. — eFigure 1. Spatial Distribution of Baseline Populations Involved in the Cohort Study eFigure 2. Flowchart for the Study Participants Selection eFigure 3. The Constructed Directed Acyclic Graph for the Potential Confounders eFigure 4. Results of Subgroup Analyses for the Associations Between EPDS Scores (per IQR Increase) With Fetal FL and AC Growth Rates eTable 1. Likelihood Ratio Test for Adjusted Models With and Without Random Slope of Gestational Age at Ultrasonography Measurement eTable 2. Results of the Multivariable Adjusted Analyses of Quintiles of Maternal EPDS Score With Fetal Growth Rates eTable 3. Akaike Information of the Generalized Additive Models for Location, Scale, and Shape eTable 4. Associations of EPDS Score (per IQR Increase) With SD Scores of Fetal Growth Parameters eTable 5. Associations Between EPDS Score (per IQR Increase) With Fetal Growth Rate, After Excluding Participants With Gestational Diabetes or Hypertensive Disorders of Pregnancy eTable 6. Associations Between EPDS Score (per IQR Increase) With Fetal Growth Rate, After Excluding Participants With Severe Disease Before Pregnancy eTable 7. Associations Between EPDS Score (per IQR Increase) With Fetal Growth Rate, After Excluding Participants With Severe Disease Before Pregnancy, Gestational Diabetes, or Hypertensive Disorders of Pregnancy eTable 8. Associations Between EPDS Score (per IQR Increase) With Fetal Growth Rate, Without Imputation of Missing Covariates eMethods 1. Calculation for Gestational Age–Adjusted SD Scores eMethods 2. Calculation for Healthy Eating Scores in Our Study [file jamanetwopen-e2346018-s001.pdf]

Supplemental Online Content

Zhang L, Li P, Ge Q, et al. Maternal prenatal depressive symptoms and fetal growth during the critical rapid growth stage. *JAMA Netw Open.* 2023;6(12):e2346018. doi:10.1001/jamanetworkopen.2023.46018

eFigure 1. Spatial Distribution of Baseline Populations Involved in the Cohort Study

eFigure 2. Flowchart for the Study Participants Selection

eFigure 3. The Constructed Directed Acyclic Graph for the Potential Confounders

eFigure 4. Results of Subgroup Analyses for the Associations Between EPDS Scores (per IQR Increase) With Fetal FL and AC Growth Rates

eTable 1. Likelihood Ratio Test for Adjusted Models With and Without Random Slope of Gestational Age at Ultrasonography Measurement

eTable 2. Results of the Multivariable Adjusted Analyses of Quintiles of Maternal EPDS Score With Fetal Growth Rates

eTable 3. Akaike Information of the Generalized Additive Models for Location, Scale, and Shape

eTable 4. Associations of EPDS Score (per IQR Increase) With SD Scores of Fetal Growth Parameters

eTable 5. Associations Between EPDS Score (per IQR Increase) With Fetal Growth Rate, After Excluding Participants With Gestational Diabetes or Hypertensive Disorders of Pregnancy

eTable 6. Associations Between EPDS Score (per IQR Increase) With Fetal Growth Rate, After Excluding Participants With Severe Disease Before Pregnancy

eTable 7. Associations Between EPDS Score (per IQR Increase) With Fetal Growth Rate, After Excluding Participants With Severe Disease Before Pregnancy, Gestational Diabetes, or Hypertensive Disorders of Pregnancy

eTable 8. Associations Between EPDS Score (per IQR Increase) With Fetal Growth Rate, Without Imputation of Missing Covariates

eMethods 1. Calculation for Gestational Age–Adjusted SD Scores

eMethods 2. Calculation for Healthy Eating Scores in Our Study

eReferences.

This supplemental material has been provided by the authors to give readers additional information about their work.

© 2023 Zhang L et al. *JAMA Network Open.*

eFigure 1. Spatial Distribution of Baseline Populations Involved in the Cohort Study

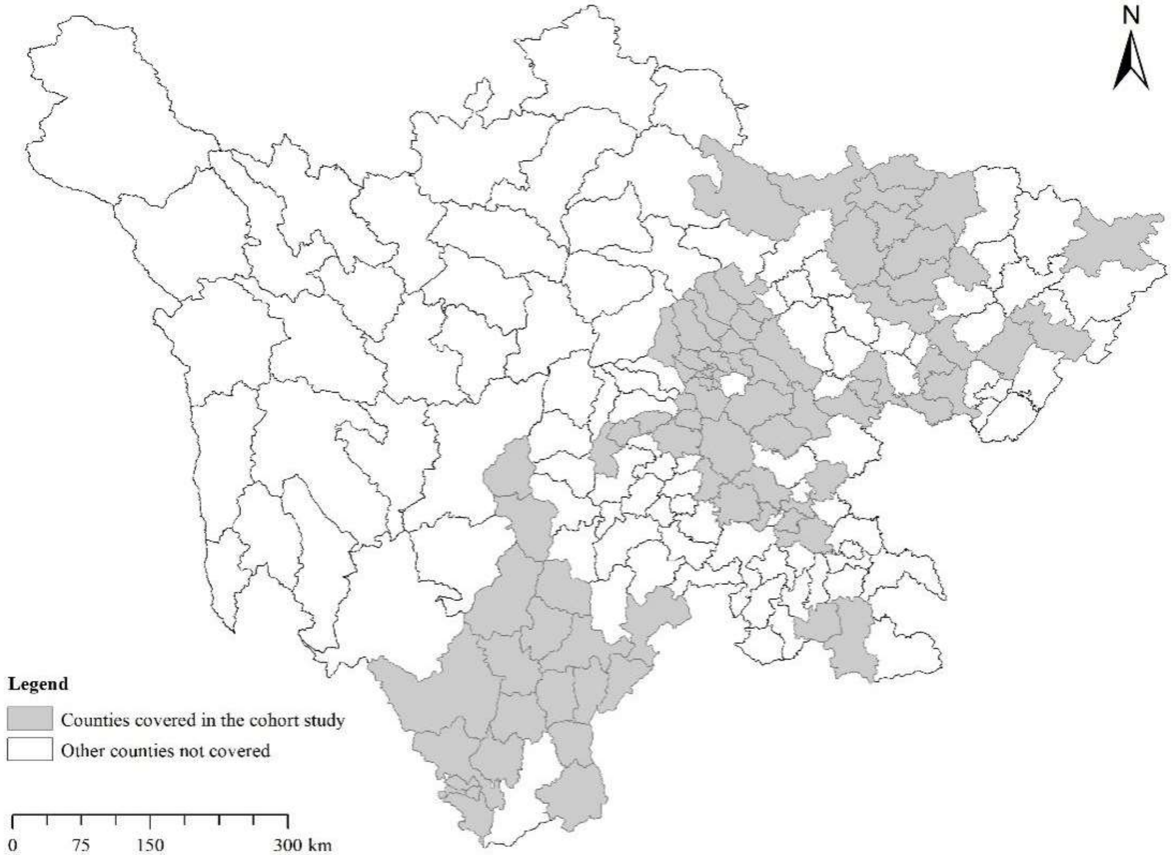

© 2023 Zhang L et al. *JAMA Network Open.*

eFigure 2. Flowchart for the Study Participants Selection

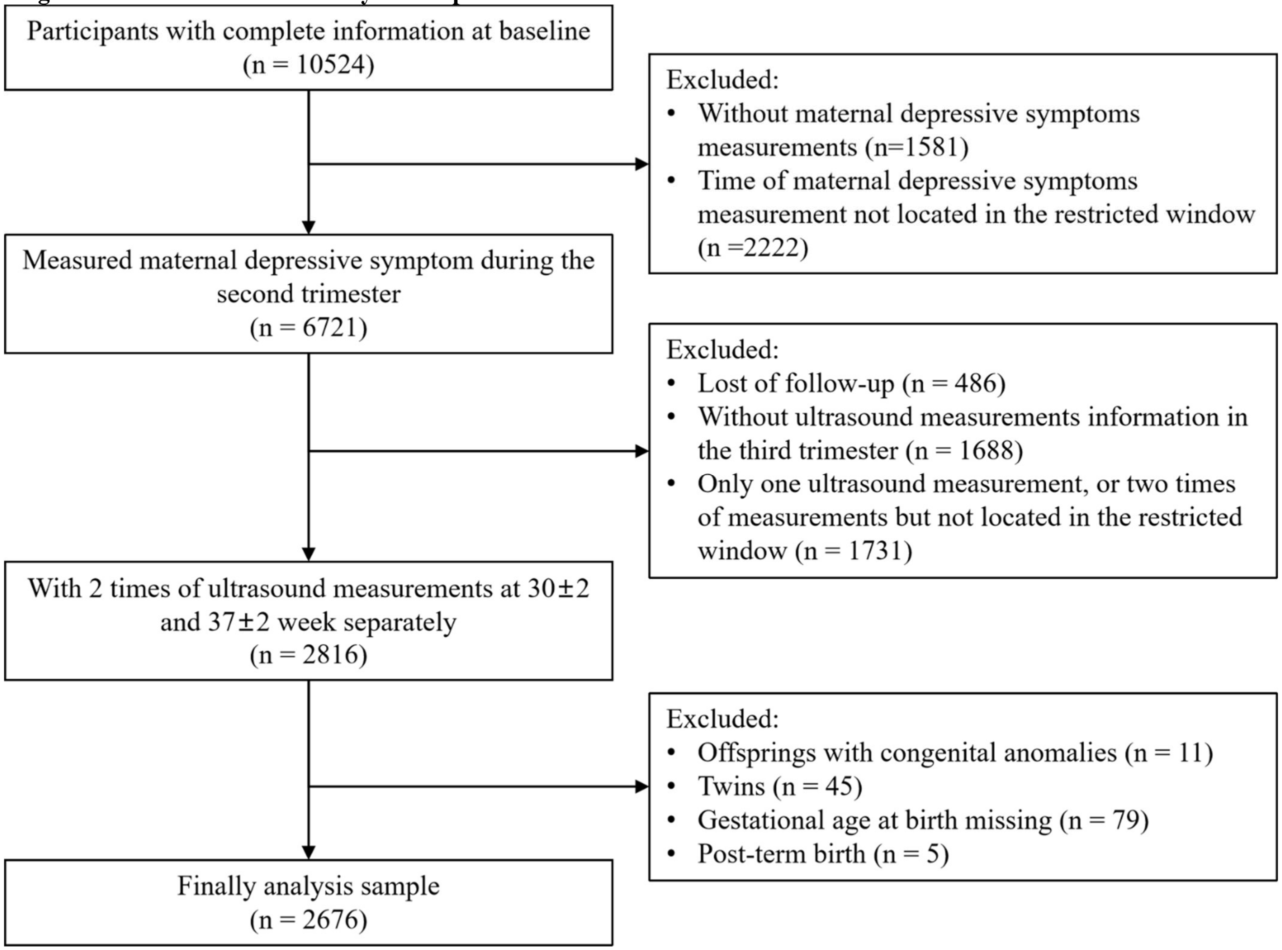

© 2023 Zhang L et al. *JAMA Network Open.*

eFigure 3. The Constructed Directed Acyclic Graph for the Potential Confounders

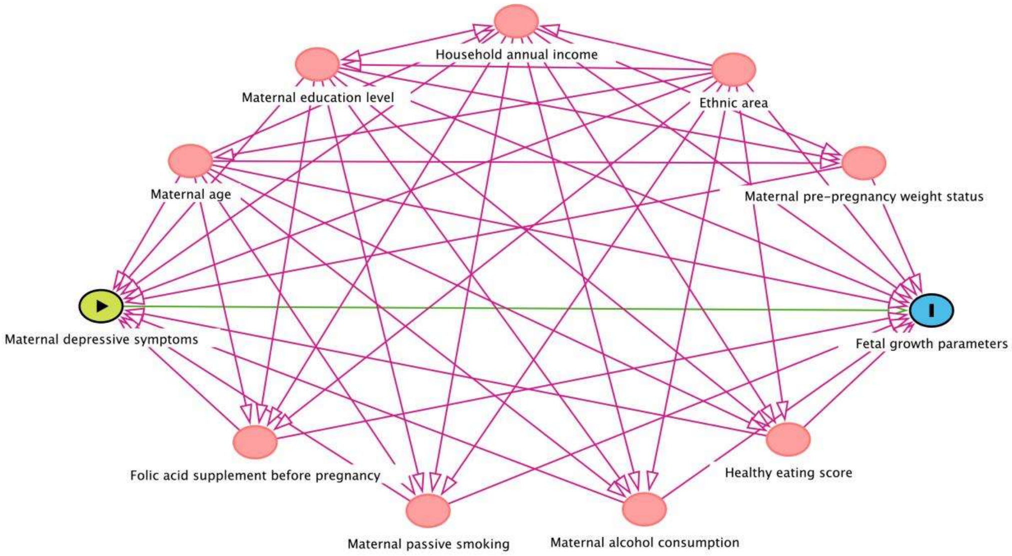

© 2023 Zhang L et al. *JAMA Network Open*.

eFigure 4. Results of Subgroup Analyses for the Associations Between EPDS Scores (per IQR Increase) With Fetal FL and AC Growth Rates

Subgroup	Participants, No(%)	β (95%CI) for FL	P for FL	β (95%CI) for AC	P for AC
Sex			0.024		< 0.001
Male	1382 (51.64)	-0.30 (-0.56, -0.04)		-1.29 (-2.66, 0.08)	
Female	1294 (48.36)	-0.55 (-0.81, -0.30)		-2.90 (-4.17, -1.63)	
Education level			0.459		0.376
Junior school and below	607 (22.68)	-0.12 (-0.46, 0.23)		-2.73 (-4.58, -0.87)	
Senior school	859 (32.10)	-0.73 (-1.07, -0.40)		-2.87 (-4.65, -1.10)	
Junior college	750 (28.03)	-0.34 (-0.69, 0.01)		-0.50 (-2.25, 1.25)	
University and above	460 (17.19)	-0.48 (-0.95, -0.01)		-2.69 (-4.96, -0.42)	
Annual household income			< 0.001		< 0.001
<60000 CNY	1136 (42.45)	-0.46 (-0.74, -0.19)		-1.76 (-3.18, -0.33)	
60000-99999 CNY	1039 (38.83)	-0.05 (-0.35, 0.25)		-0.89 (-2.41, 0.63)	
>99999 CNY	501 (18.72)	-0.79 (-1.17, -0.40)		-2.91 (-5.03, -0.79)	
Ethnic minority area			0.083		0.184
No	2311 (86.36)	-0.36 (-0.56, -0.16)		-1.87 (-2.92, -0.82)	
Yes	365 (13.64)	-0.31 (-0.84, 0.22)		-1.15 (-3.73, 1.43)	
Pre-pregnancy weight status			0.073		0.653
Underweight	484 (18.09)	-0.54 (-1.01, -0.08)		-2.42 (-4.54, -0.30)	
Normal weight	1744 (65.17)	-0.46 (-0.69, -0.24)		-2.19 (-3.37, -1.01)	
Overweight	448 (16.74)	-0.13 (-0.54, 0.28)		-1.09 (-3.34, 1.16)	
Healthy eating score			0.176		0.152
Quintile 1	568 (21.23)	-0.07 (-0.46, 0.33)		-1.81 (-3.87, 0.25)	
Quintile 2	433 (16.18)	-0.16 (-0.62, 0.30)		-1.21 (-3.65, 1.22)	
Quintile 3	499 (18.65)	-0.62 (-1.02, -0.23)		-4.67 (-6.80, -2.53)	
Quintile 4	551 (20.59)	-0.39 (-0.82, 0.04)		-1.75 (-3.76, 0.27)	
Quintile 5	625 (23.36)	-0.64 (-1.03, -0.26)		-0.96 (-2.97, 1.05)	

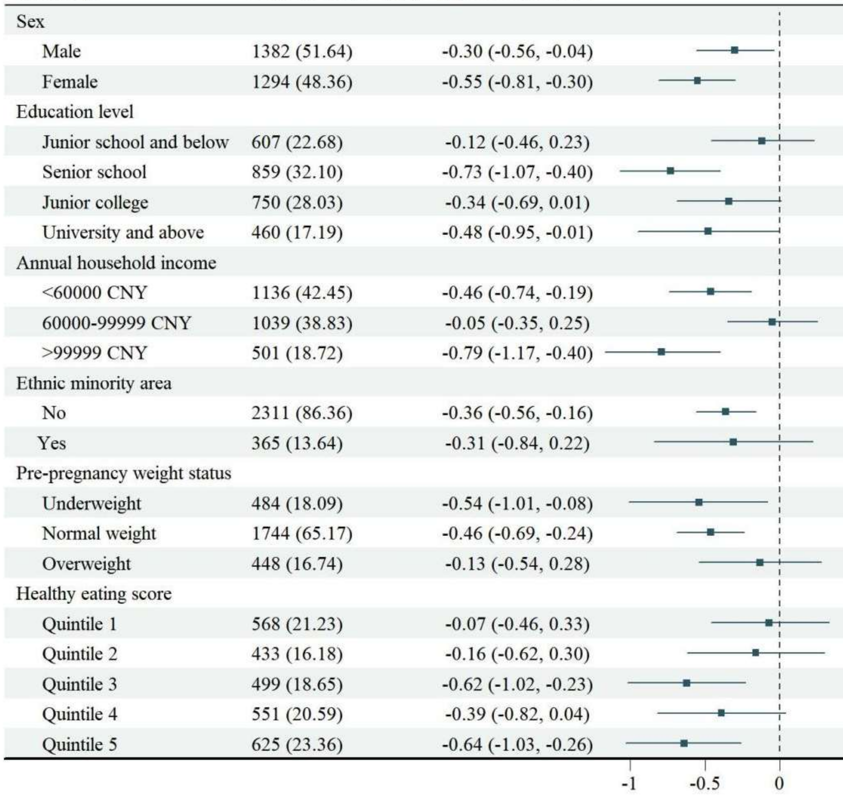

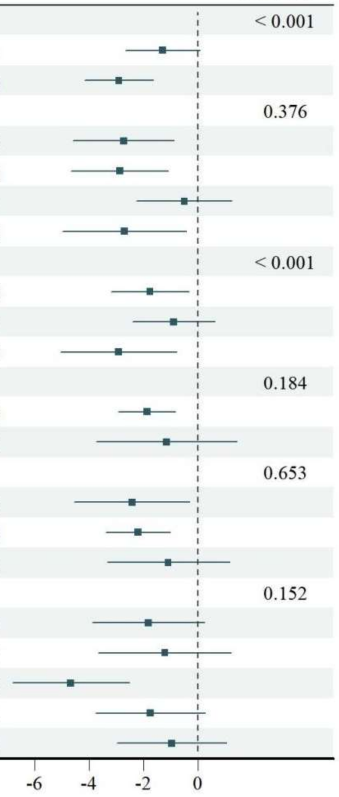

Abbreviations: FL, femur length; AC, abdominal circumference; Edinburgh postnatal depression scale; GAUM, gestational age at each ultrasound measurement.
Note: P value indicated the significance of the GAAM × EPDS scores × subgroup categories interaction term.

© 2023 Zhang L et al. *JAMA Network Open.*

eTable 1. Likelihood Ratio Test for Adjusted Models With and Without Random Slope of Gestational Age at Ultrasonography Measurement

Growth parameters	Random slope of GAUM			No random slope of GAUM			Likelihood Ratio	P Value
	df	AIC	Log likelihood	df	AIC	Log likelihood		
BPD, mm	28	28495.06	-14219.53	26	28693.48	-14320.74	202.42	<0.001
FL, mm	28	26522.25	-13233.12	26	26667.65	-13307.82	149.40	<0.001
AC, mm	28	43823.03	-21883.51	26	43822.20	-21885.10	3.18	0.204
EFW, g	28	75414.79	-37679.39	26	75686.94	-37817.47	276.15	<0.001

Abbreviations: BPD, biparietal diameter; FL, femur length; AC, abdominal circumference; EFW, estimated fetal weight; GAUM, gestational age at each ultrasound measurement.

© 2023 Zhang L et al. *JAMA Network Open.*

eTable 2. Results of the Multivariable Adjusted Analyses of Quintiles of Maternal EPDS Score With Fetal Growth Rates

Growth parameters	Quintiles of EPDS score						GAUM × Quintiles of EPDS score					
	Q1	Q2	Q3	Q4	Q5	P for trend[b]	Q1	Q2	Q3	Q4	Q5	P for trend[b]
BPD, mm						0.004						0.009
Crude	Ref	-0.32 (-0.91, 0.27)	0.27 (-0.28, 0.83)	0.04 (-0.50, 0.58)	-0.30 (-0.85, 0.24)		Ref	0.32 (-0.28, 0.91)	-0.46 (-1.02, 0.10)	-0.48 (-1.03, 0.06)	-0.50 (-1.06, 0.05)	
Adjusted[a]	Ref	-0.16 (-0.74, 0.43)	0.43 (-0.11, 0.98)	0.23 (-0.30, 0.76)	0.20 (-0.35, 0.75)		Ref	0.31 (-0.28, 0.91)	-0.45 (-1.01, 0.11)	-0.46 (-1.00, 0.09)	-0.45 (-1.00, 0.10)	
FL, mm						<0.001						<0.001
Crude	Ref	-0.26 (-0.74, 0.21)	0.10 (-0.34, 0.55)	-0.05 (-0.48, 0.38)	0.01 (-0.42, 0.45)		Ref	0.19 (-0.32, 0.71)	-0.38 (-0.87, 0.10)	**-0.54 (-1.01, -0.07)**	**-0.86 (-1.33, -0.38)**	
Adjusted[a]	Ref	-0.26 (-0.73, 0.21)	0.13 (-0.31, 0.57)	0.03 (-0.40, 0.46)	0.22 (-0.22, 0.66)		Ref	0.19 (-0.33, 0.70)	-0.37 (-0.85, 0.11)	**-0.52 (-0.99, -0.05)**	**-0.81 (-1.28, -0.33)**	
AC, mm						<0.001						<0.001
Crude	Ref	-0.31 (-2.40, 1.77)	**-2.43 (-4.39, -0.46)**	-0.69 (-2.60, 1.21)	0.31 (-1.62, 2.24)		Ref	1.31 (-1.33, 3.95)	**2.52 (0.03, 5.01)**	-0.49 (-2.91, 1.92)	**-3.33 (-5.77, -0.88)**	
Adjusted[a]	Ref	-0.27 (-2.33, 1.80)	**-2.11 (-4.06, -0.16)**	-0.1 (-1.99, 1.79)	1.37 (-0.58, 3.31)		Ref	1.29 (-1.35, 3.93)	**2.57 (0.08, 5.05)**	-0.39 (-2.80, 2.02)	**-3.10 (-5.55, -0.66)**	
EFW, g						<0.001						<0.001
Crude	Ref	-14.67 (-50.04, 20.7)	-20.69 (-54.04, 12.66)	-9.74 (-42.03, 22.56)	-1.74 (-34.46, 30.98)		Ref	27.27 (-24.6, 79.14)	4.43 (-44.48, 53.34)	-42.19 (-89.54, 5.17)	**-94.92 (-142.91, -46.93)**	
Adjusted[a]	Ref	-12.38 (-47.33, 22.56)	-14.48 (-47.53, 18.57)	2.43 (-29.59, 34.45)	22.78 (-10.38, 55.95)		Ref	26.71 (-25.07, 78.5)	5.32 (-43.51, 54.15)	-40.16 (-87.44, 7.13)	**-90.56 (-138.53, -42.59)**	

Abbreviations: BPD, biparietal diameter; FL, femur length; AC, abdominal circumference; EFW, estimated fetal weight; EPDS, Edinburgh postnatal depression scale; GAUM, gestational age at each ultrasound measurement.

[a] Models were adjusted for maternal age, ethnic area, maternal education level, household annual income, maternal pre-pregnancy weight status, parity, folic acid supplement before pregnancy, maternal passive smoking, maternal alcohol consumption, healthy eating score, gestational age at each ultrasound measurement and interaction terms between GAUM and passive smoking and alcohol consumption.

[b] Calculated using the median of each quintile as a continuous variable.

© 2023 Zhang L et al. *JAMA Network Open.*

eTable 3. Akaike Information of the Generalized Additive Models for Location, Scale, and Shape

Growth parameters	Distributions		
	**BCPE**	**BCCG**	**BCT**
28 - 32 week			
Biparietal diameter	**2302.61**	2330.26	2304.45
Femur length	884.61	1070.68	**856.42**
Abdominal circumference	8299.89	8384.28	**8285.05**
Estimated fetal weight	**35867.52**	35881.70	35868.82
35 - 39 week			
Biparietal diameter	942.52	1020.35	**929.87**
Femur length	-192.25	-59.78	**-223.23**
Abdominal circumference	9096.28	9204.91	**9081.15**
Estimated fetal weight	37945.00	37987.65	**37927.58**

Abbreviations: BCPE, box-cox power exponential; BCCG, box-cox cole-green; BCT, box-cox t.

© 2023 Zhang L et al. *JAMA Network Open.*

eTable 4. Associations of EPDS Score (per IQR Increase) With SD Scores of Fetal Growth Parameters

Growth parameters	Crude β (95% CI)		Adjusted β (95% CI) [a]	
	EPDS score	GAUM×EPDS score	EPDS score	GAUM×EPDS score
BPD, SD	-0.04 (-0.10, 0.01)	-0.06 (-0.11, -0.01)	-0.03 (-0.35, 0.29)	-0.05 (-0.10, 0.00)
FL, SD	-0.02 (-0.07, 0.03)	-0.12 (-0.17, -0.07)[b]	-0.05 (-0.40, 0.30)	-0.12 (-0.17, -0.06) [b]
AC, SD	-0.01 (-0.06, 0.04)	-0.07 (-0.12, -0.01)	0.07 (-0.28, 0.43)	-0.06 (-0.12, -0.01)
EFW, SD	-0.02 (-0.08, 0.03)	-0.09 (-0.15, -0.04) [b]	0.01 (-0.08, 0.09)	-0.09 (-0.14, -0.04) [b]

Abbreviations: BPD, biparietal diameter; FL, femur length; AC, abdominal circumference; EFW, estimated fetal weight; EPDS, Edinburgh postnatal depression scale; GAUM, gestational age at each ultrasound measurement.

[a] Adjusted for maternal age, ethnic area, maternal education level, household annual income, maternal pre-pregnancy weight status, folic acid supplement before pregnancy, parity, maternal passive smoking, maternal alcohol consumption, healthy eating score, gestational age at each ultrasound measurement and interaction terms between GAUM and passive smoking and alcohol consumption.

[b] Indicates a significant association after a false discovery rate correction.

© 2023 Zhang L et al. *JAMA Network Open.*

eTable 5. Associations Between EPDS Score (per IQR Increase) With Fetal Growth Rate, After Excluding Participants With Gestational Diabetes or Hypertensive Disorders of Pregnancy (n = 2459)

Growth parameters	EPDS score		GAUM×EPDS score	
	**β (95% CI) [a]**	**P**	**β (95% CI) [a]**	**P**
Biparietal diameter, mm	-0.99 (-2.44, 0.45)	0.982	-0.16 (-0.38, 0.05)	0.139
Femur length, mm	-0.68 (-1.93, 0.57)	0.363	-0.40 (-0.59, -0.21)	< 0.001[b]
Abdominal circumference, mm	-1.98 (-8.41, 4.45)	0.010	-1.86 (-2.83, -0.89)	< 0.001[b]
Estimated fetal weight, g	-60.52 (-186.58, 65.55)	0.049	-47.97 (-66.96, -28.97)	< 0.001 [b]

Abbreviations: EPDS, Edinburgh postnatal depression scale; GAUM, gestational age at each ultrasound measurement.

[a] Models were adjusted for maternal age, ethnic area, maternal education level, household annual income, maternal pre-pregnancy weight status, parity, folic acid supplement before pregnancy, maternal passive smoking, maternal alcohol consumption, healthy eating score, gestational age at each ultrasound measurement and interaction terms between GAUM and passive smoking and alcohol consumption.

[b] Indicates a significant association after a false discovery rate correction.

© 2023 Zhang L et al. *JAMA Network Open.*

eTable 6. Associations Between EPDS Score (per IQR Increase) With Fetal Growth Rate, After Excluding Participants With Severe Disease Before Pregnancy (n = 2640).

Growth parameters	EPDS score		GAUM×EPDS score	
	β (95% CI) [a]	P	β (95% CI) [a]	P
Biparietal diameter, mm	-0.72 (-2.13, 0.69)	0.318	-0.20 (-0.41, 0.01)	0.068
Femur length, mm	-0.65 (-1.86, 0.57)	0.296	-0.39 (-0.58, -0.21)	< 0.001[b]
Abdominal circumference, mm	-0.98 (-7.26, 5.29)	0.758	-1.96 (-2.90, -1.02)	< 0.001[b]
Estimated fetal weight, g	-39.70 (-162.62, 83.22)	0.527	-49.78 (-68.26, -31.31)	< 0.001[b]

Abbreviations: EPDS, Edinburgh postnatal depression scale; GAUM, gestational age at each ultrasound measurement.
Note: Severe disease included hypertension, heart disease, hyperthyroidism, hypothyroidism, depression and schizophrenia.
[a] Models were adjusted for maternal age, ethnic area, maternal education level, household annual income, maternal pre-pregnancy weight status, parity, folic acid supplement before pregnancy, maternal passive smoking, maternal alcohol consumption, healthy eating score, gestational age at each ultrasound measurement and interaction terms between GAUM and passive smoking and alcohol consumption.
[b] Indicates a significant association after a false discovery rate correction.

© 2023 Zhang L et al. *JAMA Network Open.*

eTable 7. Associations Between EPDS Score (per IQR Increase) With Fetal Growth Rate, After Excluding Participants With Severe Disease Before Pregnancy, Gestational Diabetes, or Hypertensive Disorders of Pregnancy (n = 2429).

Growth parameters	EPDS score		GAUM×EPDS score	
	β (95% CI) [a]	P	β (95% CI) [a]	P
Biparietal diameter, mm	-0.74 (-2.23, 0.75)	0.332	-0.16 (-0.38, 0.06)	0.151
Femur length, mm	-0.40 (-1.69, 0.89)	0.543	-0.40 (-0.59, -0.21)	< 0.001[b]
Abdominal circumference, mm	-0.31 (-6.94, 6.33)	0.927	-1.86 (-2.84, -0.89)	< 0.001[b]
Estimated fetal weight, g	-26.07 (-155.92, 103.78)	0.694	-47.8 (-66.89, -28.71)	< 0.001[b]

Abbreviations: EPDS, Edinburgh postnatal depression scale; GAUM, gestational age at each ultrasound measurement.

Note: Severe disease included hypertension, heart disease, hyperthyroidism, hypothyroidism, depression and schizophrenia.

[a] Models were adjusted for maternal age, ethnic area, maternal education level, household annual income, maternal pre-pregnancy weight status, parity, folic acid supplement before pregnancy, maternal passive smoking, maternal alcohol consumption, healthy eating score, gestational age at each ultrasound measurement and interaction terms between GAUM and passive smoking and alcohol consumption.

[b] Indicates a significant association after a false discovery rate correction.

© 2023 Zhang L et al. *JAMA Network Open.*

eTable 8. Associations Between EPDS Score (per IQR Increase) With Fetal Growth Rate, Without Imputation of Missing Covariates (n = 2262).

Growth parameters	EPDS score		GAUM×EPDS score	
	β (95% CI) [a]	P	β (95% CI) [a]	P
Biparietal diameter, mm	-0.62 (-2.01, 0.76)	0.378	-0.05 (-0.27, 0.17)	0.636
Femur length, mm	-0.95 (-2.11, 0.20)	0.105	-0.23 (-0.41, -0.05)	0.011[b]
Abdominal circumference, mm	-2.10 (-8.08, 3.88)	0.492	-1.07 (-2.01, -0.13)	0.026
Estimated fetal weight, g	-64.12 (-181.18, 52.93)	0.283	-29.2 (-47.58, -10.82)	0.002[b]

Abbreviations: EPDS, Edinburgh postnatal depression scale; GAUM, gestational age at each ultrasound measurement.

[a] Models were adjusted for maternal age, ethnic area, maternal education level, household annual income, maternal pre-pregnancy weight status, parity, folic acid supplement before pregnancy, maternal passive smoking, maternal alcohol consumption, healthy eating score, gestational age at each ultrasound measurement and interaction terms between GAUM and passive smoking and alcohol consumption.

[b] Indicates a significant association after a false discovery rate correction.

© 2023 Zhang L et al. *JAMA Network Open.*

eMethods 1. Calculation for Gestational Age–Adjusted SD Scores

We calculated the gestational age-adjusted SD scores using GAMLSS package (version 5.4-12) in R software (version 4.1.1) [1,2]. Each fetal growth parameter was modeled by cubic splines of gestational age based on different distribution families including the Box-Cox power exponential, Box-Cox Cole-Green and Box-Cox t families[3]. The Akaike's information criteria was used to select the best fitting model for each fetal growth parameter (eTable 3). Finally, the SD scores of each fetal growth parameter were generated by the best fitting model, which represents the percentile of fetal size for a specific gestational age.

© 2023 Zhang L et al. *JAMA Network Open*.

eMethods 2. Calculation for Healthy Eating Scores in Our Study

Dietary intake was investigated during the second trimester by a semi-quantitative validated food frequency questionnaire. Dietary scores were calculated using collected diet data similar to the Alternative Health Eating Index-2010 [4]. We included 7 healthy foods (cereals, fresh vegetables, fruits, fish, eggs, soybeans and nuts) and 5 unhealthy foods (red meat, poultry, fired foods, processed meats and pickled vegetables) into consideration. The dietary score of each food subgroup was based on the quintiles of daily intake frequency, with health foods scored from 1 to 5 and unhealth foods scored from 5 to 1. The total dietary score was the sum of scores for each food subgroup on a scale from 1 to 60, with higher dietary score representing a better diet.

© 2023 Zhang L et al. *JAMA Network Open.*

eReferences

1. Pitt D, Trück S, van den Honert R, Wong WW. Modeling risks from natural hazards with generalized additive models for location, scale and shape. *J Environ Manage*. 2020;275:111075. doi:10.1016/j.jenvman.2020.111075

2. Rigby RA, Stasinopoulos DM. Generalized Additive Models for Location, Scale and Shape. *Journal of the Royal Statistical Society Series C: Applied Statistics*. 2005;54(3):507-554. doi:10.1111/j.1467-9876.2005.00510.x

3. Hu J, Peng Y, Zheng T, Zhang B, Liu W, Wu C, et al. Effects of trimester-specific exposure to vanadium on ultrasound measures of fetal growth and birth size: a longitudinal prospective prenatal cohort study. *Lancet Planet Health*. 2018;2(10):e427-e437. doi:10.1016/s2542-5196(18)30210-9

4. Chiuve SE, Fung TT, Rimm EB, Hu FB, McCullough ML, Wang M, et al. Alternative dietary indices both strongly predict risk of chronic disease. *J Nutr*. 2012;142(6):1009-1018. doi:10.3945/jn.111.157222

© 2023 Zhang L et al. *JAMA Network Open*.
