# Supplementary material for: Maternal Prenatal Depressive Symptoms and Fetal Growth During the Critical Rapid Growth Stage
Source: JAMA Netw Open. 2023 Dec 4;6(12):e2346018. doi: 10.1001/jamanetworkopen.2023.46018 (PMC10696489; doi:10.1001/jamanetworkopen.2023.46018)
Supplement: Supplement 2. — Data Sharing Statement [file jamanetwopen-e2346018-s002.pdf]

Data Sharing Statement

Zhang. Maternal Prenatal Depressive Symptoms and Fetal Growth During the Critical Rapid Growth Stage. *JAMA Netw Open*. Published December 04, 2023.
doi:10.1001/jamanetworkopen.2023.46018

Data
Data available: No

Additional Information
Explanation for why data not available: Data in this study contains sensitive information that cannot be shared through public deposition due to information management restrictions to protect individual's privacy. External researchers outside of this study team are not allowed to access personal data. Access to data will only be granted upon request to the project team and ethics committee.
